# Dietary Protein Intake and Its Associations With Bone Properties Using Peripheral Quantitative Computed Tomography and Dual-Energy X-Ray Absorptiometry in Endurance-Trained Individuals

**DOI:** 10.1016/j.cdnut.2025.107459

**Published:** 2025-05-09

**Authors:** Silar Gardy, Ada Sevinc, Jennifer Levee, Sofia V Ferreira, Julia-Rose Linardatos, Andrea R Josse, Tyler A Churchward-Venne, Jenna C Gibbs

**Affiliations:** 1 Department of Kinesiology and Physical Education, McGill University, Montréal QC, Canada; 2 Department of Kinesiology and Health Sciences, University of Waterloo, Waterloo ON, Canada; 3 School of Kinesiology and Health Science, York University, Toronto ON, Canada; 4 Muscle Health Research Centre, Faculty of Health, York University, Toronto ON, Canada; 5 Metabolic Disorders and Complications Program, The Research Institute of the McGill University Health Centre, Montréal QC, Canada; 6 Division of Geriatric Medicine, McGill University, Montréal QC, Canada

**Keywords:** endurance athletes, protein intake, bone mineral density, dual-energy x-ray absorptiometry, peripheral quantitative computed tomography, bone health

## Abstract

**Background:**

Endurance athletes are at greater risk of compromised bone health due to elevated nutritional demands and high-volume training. Optimal nutritional intake is fundamental to support athlete bone health, and dietary protein is an essential nutrient for the maintenance of bone and muscle tissue. Studies of associations between dietary protein intake and advanced imaging-based measures of bone and muscle health in endurance athletes are limited.

**Objectives:**

To examine the relationships between dietary protein intake and volumetric bone mineral density (vBMD), estimated bone strength (SSI_p_ and BSI), areal BMD (aBMD), and muscle density, cross-sectional area (CSA), and strength in male and female endurance-trained individuals.

**Methods:**

Fifty healthy young endurance-trained adults completed one-time measures. Peripheral quantitative computed tomography (pQCT) scans assessed tibial trabecular and cortical vBMD, BSI, SSI_p_, and calf muscle density and CSA. Dual-energy X-ray absorptiometry scans measured aBMD at the lumbar spine (LS) and proximal femur. Dietary protein intake (grams per kilogram of body mass per day) was calculated from 3-day 24-h dietary recalls.

**Results:**

Bivariate analyses found no correlations between total dietary protein intake and pQCT-derived bone and muscle measures. However, protein intake from animal products was correlated with SSI_p_ at the 38% (r = 0.39, *P* = 0.008) and 66% site (r = 0.44, *P* = 0.002), cortical vBMD (r = −0.34, *P* = 0.02) at the 66% site, and calf muscle CSA (r = 0.57, *P* <.001). Adjusted regression analyses revealed that higher total dietary protein intake was associated with higher LS aBMD (β = 0.398, *P* = 0.009).

**Conclusions:**

Our findings suggest that there are no relationships between total dietary protein intake and pQCT measures in endurance-trained individuals. However, positive relationships were found with protein intake from animal products and tibial SSI_p_ and muscle CSA. Additionally, our results suggest total dietary protein intake explains a small variance in LS aBMD. A future larger-scale analysis would benefit from stratifying associations by sex.

## Introduction

Endurance athletes (i.e., long-distance runners) commonly present with impaired bone health and are at higher risk of bone stress injury due to low magnitude, repetitive loading cycles, and high-volume training [[1], [2], [3]]. Endurance-based sports also necessitate large amounts of energy due to prolonged, high-intensity exercise training, and athletes often practice dietary energy restriction (both intentionally and unintentionally) [4]. This combination of training characteristics and eating behaviors results in high susceptibility to low energy availability and subsequent consequences to bone health, including lower areal and volumetric bone mineral density (aBMD, vBMD)[[5], [6], [7]]. Reports suggest long-distance runners are at increased risk of stress fractures, accounting for 15%–20% of musculoskeletal-related injuries, and long-distance runners exhibit lower whole-body and site-specific (lumbar spine [LS], femoral neck [FN]) aBMD than athletes engaged in higher-impact loading-based sports (e.g., soccer, gymnastics) [3, [8], [9], [10], [11]]. Female athletes particularly have a higher prevalence of low BMD and bone stress injury in the presence of low energy availability and/or menstrual disturbances (the Female Athlete Triad) [4]; however, male endurance athletes can also exhibit impaired bone health [12,13]. As a result, endurance athletes experience time lost from training and competition and future risk of bone fragility (i.e., low BMD for age, osteoporosis). Nutritional intake is an important modifiable factor that significantly influences the development and maintenance of bone mass [14,15]. Dietary protein is essential for musculoskeletal recovery and health, and its positive associations with bone health are well established in the general population, but are limited in athletic populations [[16], [17], [18]].

Numerous studies have examined the relationship between dietary patterns/intake and bone health-related outcomes in athletes, and several suggest the intake of dairy foods (particularly milk) has a protective effect on whole-body and site-specific aBMD and stress fracture incidence; however, these are not unanimous findings [[19], [20], [21], [22], [23]]. Although dairy products (e.g., milk, yogurt, cheese) are rich in critical bone-supporting nutrients, specifically calcium and vitamin D, they are also high in protein, which is positively associated with bone strength, BMD, and reduced fracture risk [24,25]. Contrary evidence has been reported, for instance, Barron et al. [23] found an inverse association between vegetable protein intake and lumbar spine (LS) aBMD *Z*-scores in young female endurance athletes with oligomenorrhea, even after adjusting for total calorie intake, lean mass, and body fat. However, a recent study found that a non-healthy dietary pattern (low in protein and fat) was associated with a lower aBMD *Z*-score of the LS and FN in male amateur runners [26]. Notably, Nieves et al. [20] prospectively reported that a higher intake of animal protein relative to body mass is associated with a significant annual gain in whole-body BMD and bone mineral content (BMC) in female long-distance runners, particularly those with menstrual irregularities. Further, a 6-month investigation demonstrated that a high protein diet 2.5 times the recommended daily allowance (RDA) (≥2.2 g/kg BM/d) did not pose any harmful effect on whole-body aBMD nor LS aBMD in exercise-trained females [27]. These findings and others (see Dolan [25] for a review) support the refuted notion that high intake of dietary protein may be deleterious to bone health [28,29], but also suggest that the evidence on the relationship between protein intake and bone health in athletes remains inconclusive. The joint position statement of the Academy of Nutrition and Dietetics, Dietitians of Canada, and the American College of Sports Medicine (ACSM) recommends athletes engaging in resistance and endurance exercise training to consume between 1.2 and 2.0 g/kg BM/d of protein [30]. Recent evidence in male and female endurance-trained athletes suggests consuming upward of ∼1.8 g/kg BM/d is necessary to meet whole-body requirements for dietary protein [31,32]. Many evaluations of dietary intake in endurance athletes report protein intake met ACSM recommendations [[33], [34], [35]]; however, a recent study revealed that 29% and 35% of male and female collegiate long-distance runners were not [36].

Many previous studies use dual-energy x-ray absorptiometry (DXA) to measure aBMD, however, measures of “true” vBMD and estimated bone strength (i.e., polar strength-strain index [SSI_p_], bone strength index [BSI]) derived from peripheral quantitative computed tomography (pQCT) provide more robust surrogates of “bone quality” and are better associated with athlete bone health and bone stress injury risk [2,12,[37], [38], [39]]. Muscle strength also influences lower limb bone quality, but few studies have investigated the relationships between nutritional intake and bone and muscle health parameters together in endurance athletes [1]. Dietary protein is proposed to influence bone by optimizing insulin-like growth factor-1, enhancing intestinal calcium absorption, increasing bone collagen synthesis, and suppressing markers of bone resorption and related pathways (favoring bone formation) (e.g., receptor activator of nuclear factor-kappa B) [[40], [41], [42], [43]]. It may also indirectly benefit bone health by increasing muscle size and strength, and related contractile forces acting on bone [44].

Given the positive influence of dietary protein on musculoskeletal health and the interaction between nutrition, exercise, and bone health, it is important to understand the influence of dietary protein intake on bone and muscle properties in male and female endurance athletes. Our primary objective was to examine the relationships between dietary protein intake and vBMD and estimated bone strength (SSI_p_/BSI) measured by pQCT in endurance-trained individuals. Second, we evaluated the relationships between dietary protein intake and aBMD and muscle area, density, and strength. Although we report secondary findings from a hypothesis-generating and exploratory study, based on the supporting evidence of the influence of dietary protein intake on bone health, we anticipated a positive relationship between dietary protein intake and bone outcomes from pQCT and DXA.

## Methods

### Ethics statement

This study was conducted in accordance with the 1975 Declaration of Helsinki as revised in 1983, and the protocol was approved by the McGill University Health Centre Research Ethics Board (REB # 2022-7839). The study purpose, procedures, and potential risks were communicated to participants in a virtual screening before obtaining written informed consent before the first study visit. Participants were recruited from the varsity athlete community at Montréal-based universities and colleges, local Montréal running/triathlete clubs via social media, flyers, and public announcements on university study recruitment websites. Data collection took place from March 2022 to August 2024.

### Participants

Fifty (*n* = 19 females and *n* = 31 males) individuals were eligible to participate and enrolled in the study. Participants either competed on an endurance sports team (i.e., middle-distance or long-distance running, triathlon) and/or participated in weight-bearing endurance exercise (i.e., running) >180 min/wk for the past 6 mo. Inclusion criteria for females included a naturally occurring menstrual cycle (i.e., between 21 and 35 d) or those using oral contraceptive pills at the time of study enrolment. Exclusion criteria included the following: *1)* disease (e.g., uncontrolled thyroid disease) or medication known to affect bone metabolism (e.g., hormonal contraception use other than oral contraceptives within last 3 mo before study participation, glucocorticoids); *2)* orthopedic or musculoskeletal injury/disease that limits the capacity to exercise; *3)* current smoker or tobacco user; *4)* current diagnosis of an eating disorder; or *5)* pregnant or breastfeeding.

### Study design

This study reports the results from the analysis of secondary objectives from an observational, cross-sectional study designed to investigate the associations between muscle factors and bone strength, BMD, and macro-architecture measured by pQCT in endurance-trained individuals. Participants were asked to attend 2 in-person study visits, the first at the Centre for Innovative Medicine (CIM) at the McGill University Health Centre and the second at McGill University (Montréal QC). At study visit 1, participants underwent pQCT and DXA imaging scans after having abstained from caffeine and calcium supplements for ≥3 h. After the imaging tests, participants filled out a series of questionnaires using the REDCap web-based system with the assistance of a research assistant, including a demographic and health history questionnaire, Bone-Specific Physical Activity Questionnaire (BPAQ), Low Energy Availability in Females Questionnaire (LEAF-Q), Eating Disorder Inventory-3 (EDI-3), and the Three Factor Eating Questionnaire (TFEQ). At study visit 2, participants arrived after having abstained from caffeine, strenuous exercise, and alcohol for ≥3 h and completed a series of performance-based tests of upper- and lower-limb muscle strength and peak aerobic capacity. Additionally, participants were asked in between study visits to: *1)* wear a triaxial accelerometer on their waist for 7 consecutive days; *2)* wear a heart monitor during every training session in the 7-day period; and *3)* complete a 24-h dietary recall using a web-based tool over 3 d. Eleven participants attended the study visits in reverse due to scheduling conflicts.

#### Dietary assessment

Dietary intake data were collected and analyzed using the Automated Self-Administered 24-hour Dietary Assessment Tool (ASA24; National Cancer Institute) (https://epi.grants.cancer.gov/asa24) version 2020 (44 participants) and 2022 (6 participants), a validated measure of 24-h food recall [45]. Participants completed 3 24-h dietary recalls (2 weekdays, 1 weekend day) to capture the participants’ usual diet. Recalls were verified by a single research team member, and while participants were asked to record recalls on training days, this was not confirmed. The mean total intake for dietary energy, macronutrients, and micronutrients was calculated for males and females and compared with recommended values for athletes, the acceptable macronutrient distribution range (AMDR), and the RDA [30,46]. To analyze protein intake by source (i.e., animal), the variable coded “PF_MPS_TOTAL” from participants’ recalls was extracted and defined by ASA24 as “Total of meat, poultry, seafood, organ meat, and cured meat (oz. eq.)” (only 46 participants were included in this analysis due to incomplete recalls).

#### vBMD, muscle area and density, and estimated bone strength

A trained bone densitometry technologist performed pQCT scans at the tibia using the XCT 3000 scanner (Stratec Medizintechnik). pQCT acquisition parameters were 2.5 mm slice thickness, 0.5 × 0.5 mm in-plane pixel size, and a tube voltage of 60 kV operated at 0.3 mA. Images were analyzed using the Stratec software (Orthometrix Inc) to derive the following variables at the 4%, 38%, and/or 66% sites of the tibia (measured from the distal end of the medial malleolus to the proximal end of the medial tibia plateau): trabecular and cortical vBMD and area, and estimated bone strength (SSI and BSI). We selected sites that represent primarily trabecular bone (4%), cortical bone (38% and 66%), and maximal muscle cross-sectional area (CSA) and density in the leg (66%). Trabecular vBMD and area were analyzed from the 4% site using the CALCBD analysis—contour mode 1 with a threshold of 180 mg/cm^3^ and a trabecular area of 45%. Cortical vBMD and area were analyzed at the 38% and 66% sites using the CORTBD analysis—contour mode 1 and a threshold of 710 mg/cm^3^. SSI_p_ was analyzed at the 66% site using a threshold of 280 mg/cm^3^. At the 4% site, BSI was calculated using the equation: BSI = total bone cross-sectional area (ToA, mm^2^)∗(total bone volumetric bone mineral density (ToD mg·cm^3^)^2^) as a measure of bone strength to resist compressive forces at the end of long bones [47]. Calf muscle CSA was analyzed at the 66% site using a threshold of 280 mg/cm^3^ with contour mode 1. Segmentation of muscle from subcutaneous fat was analyzed using a threshold of 40 mg/cm^3^ with contour mode 3. At the 66% site, muscle CSA was calculated using the equation: ToA − ToA + muscle area. Muscle density was calculated by dividing total muscle mass by muscle CSA [48].

#### aBMD and body composition

DXA (Lunar Prodigy DXA, GE Healthcare) was used to determine whole-body and site-specific aBMD and body composition. LS, total hip (TH), and femoral neck (FN) aBMD were determined from the appendicular spine and proximal left femur scans. Whole-body aBMD and body composition measures were determined from a whole-body scan. All measures were determined using the GE Lunar iDXA software (version 15.0). Scans were performed and analyzed by a trained technician after daily calibration. BMI was calculated as kg/m^2^ from the height and body mass measurements taken without shoes while wearing light clothing.

#### Questionnaires

Participants completed a demographic and health history questionnaire to obtain information about variables of interest, including age, sex, self-identified gender, race/ethnicity, medication and supplement use, weight change patterns, and history of disease, illness, and musculoskeletal recovery. This questionnaire also obtained information on training history and primary and secondary endurance sport participation: *1)* middle-distance running (800 m–3000 m); *2)* long-distance running (3000 m–marathon); *3)* triathlon; *4)* swimming; *5)* cycling; and *6)* other. The BPAQ was used to assess participation in bone-specific physical activity and measure self-reported lifetime physical activity (type, age, years of participation) and type and frequency of participation in the past 12 months [49]. Responses were analyzed using algorithms and effective load ratings [49]. The LEAF-Q was used to assess risk for low energy availability, but only females responded to questions regarding menstrual function, history of menstrual irregularities, and contraceptive use [50]. The LEAF-Q for males (LEAM-Q) was not used as it was not a validated tool at the time of study delivery [51]. The TFEQ-revised 21-item version includes questions on a 4-point Likert scale and was completed for the dietary cognitive restraint subscale, which is commonly used for athletes to assess disordered eating [52]. The score from each item was recoded and summed to generate a subscale score, which ranged from 0 to 100. The cognitive restraint subscale was used to assess the extent to which an individual controls their food intake to maintain or lose body weight, and a higher score has been associated with lower energy intake [53,54]. Drive for Thinness, Body Dissatisfaction, and Bulimia subscales from the EDI-3 were used to measure disordered eating attitudes and behaviors. The Drive for Thinness subscale ranges from 0 to 28 and measures the presence of an excessive concern with dieting, preoccupation with weight, and an extreme pursuit of thinness. The Body Dissatisfaction and Bulimia subscale scores range from 0 to 40 and 0 to 32, respectively. All 3 subscale raw scores were recoded and summed to generate an EDI-3 total score ranging from 0 to 100, with a higher score reflecting a higher eating disorder risk [55]. The EDI-3 was not developed for and validated in active and/or athletic populations; however, these 3 subscales are commonly used together to assess disordered eating attitudes and behaviors in athletes at risk of Female and Male Athlete Triad conditions [53,56]. Reliability and validity of the EDI-3 have been established in individuals aged 13–53 years [55,57].

#### Muscle strength

Upper-body muscle strength as an indicator of whole-body strength was measured with a validated isometric hand grip test by Jamar hydraulic hand dynamometer Model J00105 (Sammons Preston, Patterson Medical) [58]. The test was performed for 3 repetitions per arm, and the maximum grip strength between both limbs was reported. Lower limb muscle strength was measured with a validated isometric knee extensor test at 90° using the Biodex System 4 Pro dynamometer (Biodex Medical Instruments) [59]. Participants performed 4 contractions separated by 1 min of rest for 3 sets per leg, and maximum peak torque between both limbs was reported.

#### Cardiopulmonary exercise assessment

To measure peak aerobic capacity (VO_2peak_ in mL/kg/min), participants performed a progressive treadmill test using the modified Ästrand protocol [60]. The protocol requires the participant to maintain a constant speed although the treadmill grade increases per stage. Gas exchange was monitored continuously using a breath-by-breath indirect calorimetry system (SensorMedics Vmax Metabolic Cart, VIASYS Healthcare). The test was terminated upon volitional exhaustion and an attainment of a rating of perceived exertion score of >7 (out of 10). The VO_2peak_ during exercise was defined as the highest 10-s mean value for VO_2_ during the last 30 s of the exercise.

#### Physical activity assessment

Participants wore a commercially available accelerometer (GT3X+ monitors, ActiGraph) over the left hip for 7 consecutive days during waking hours to capture objective physical activity levels. Data were analyzed in 45 participants who wore the accelerometer for ≥4 d and ≥10 h/d and in 60-s epochs. Non-wear time was excluded if ≥60 min of continuous zeros and Freedson Adult VM3 (2011) cut points were used for light (0–2689 counts per minute; CPM), moderate (2690–6166 CPM), vigorous (6167–9642 CPM), and very vigorous (>9643 CPM) activity [61,62].

### Statistical analyses

Data analyses were conducted using SPSS software package version 29 (Armonk, NY, USA). The sample size required for the primary objective of this study was calculated using G∗Power software (version 3.1). Anticipating a moderate-to-large effect association (effect size = 0.33) between pQCT measures of muscle area and estimated bone strength (SSI_p_) (power = 0.80 and alpha = 0.05), it was determined that 50 adults were needed for the primary analysis of the main trial. Post hoc power analyses were conducted for the LS aBMD regression models using the observed effect sizes and sample size. These analyses indicated that the study was sufficiently powered (>0.80) to detect the observed effects for protein intake on LS aBMD in both unadjusted and adjusted models. Participant characteristics and primary/secondary outcomes were summarized using descriptive measures: mean (standard deviation and/or 95% confidence interval) for continuous variables and number (percentage) for categorical variables. Data were screened for statistical outliers and normal distributions using the Shapiro-Wilk test, and non-normally distributed data were square root transformed (total dairy intake). Defined as >2 standard deviations away from the mean, 3 variables exhibited ≤2 outlier values (moderate-to-vigorous physical activity [MVPA], and TH and FN aBMD). Sensitivity analyses were conducted to assess the robustness of the results, revealing no significant differences in the results with and without those data points included. Bivariate relationships between total dietary protein intake (grams per kilogram of body mass per day) and dependent variables of interest were determined by correlation analysis (Pearson correlation coefficient) for all participants combined. For the significantly correlated bivariate relationships (*P* < 0.05), multivariable linear regression analyses were performed using the enter method (all predictor variables entered into the model simultaneously) to adjust for potential confounders to determine the significance of total protein intake as a predictor of bone measures for all participants combined. Confounding variables were evaluated in univariate and multivariable regression models to determine their influence on any observed associations. Sex and lean body mass (LBM) were entered in the model at *P* < 0.05, while MVPA and calcium intake were entered regardless due to their established influence on bone health. Age was not included because our sample size had a narrow range. Observed local effect sizes (Cohen’s f^2^) were calculated such that f^2^ ≥ 0.02, f^2^ ≥ 0.15, and f^2^  ≥ 0.35 are interpreted as small, medium, and large effect sizes, respectively [63]. The current analysis is of secondary objectives; therefore, the results are hypothesis-generating/exploratory.

## Results

### Descriptive characteristics

Descriptive characteristics of participants are summarized in Table 1. Participants were 26 ± 4.9 y, weighed 69 ± 11.7 kg, and had a height of 173 ± 8.4 cm and a BMI of 23 ± 2.7 kg/m^2^. Forty-five (90%) participants’ primary endurance sport was long-distance running, and nearly half (46%) were averaging >40 km/wk of training. Approximately a third were averaging 25–40 km/wk of training, with the remaining averaging 15–25 km/wk. The mean VO_2peak_ was 56 mL/kg/min, with 20% of participants measuring above 60 mL/kg/min, indicating a well-trained cohort. Participants identified predominantly as White (70%) and Asian (14%). Only 2 females were using oral contraceptives, and 26% of females were 15 y or older at the onset of menarche. Eleven female participants (58%) reported their menses had stopped for 3 consecutive months or longer in the past. Twenty-five (50%) of all participants were taking a vitamin D supplement. The mean cognitive restraint score from the TFEQ was 32.3 ± 21.9 (out of 100), and the EDI-3 total score was 13.2 ± 12.3 (out of 100), which indicates our sample was largely not susceptible to controlling their food intake for body weight purposes or eating disorder risk.

**TABLE 1:** Descriptive characteristics of participants.

Variable	All ( = 50)	Males (*n* = 31)	Females (*n* = 19)
Age (y)	25.9 (24.6, 27.4)	25.7 (23.9, 27.4)	26.4 (24.0, 28.9)
Height (cm)	173.0 (170.6, 175.4)	178.0 (176.1, 180.0)	164.72 (162.1, 167.3)
Body mass (kg)	67.9 0 (64.7, 71.1)	73.9 (69.9, 78.0)	59.9 (56.8, 63.1)
BMI (kg/m^2^)	22.8 (22.1, 23.6)	23.3 (22.2, 24.4)	22.1 (21.1, 23.0)
BF (%)	19.9 (18.0, 21.9)	16.1 (14.1, 18.1)	26.2 (24.1, 28.2)
FFM (kg)	55.3 (52.1, 58.5)	62.0 (58.9, 65.2)	44.3 (42.3, 46.3)
LBM (kg)	52.6 (49.5, 55.6)	58.9 (55.9, 61.9)	42.2 (40.1, 44.2)
VO_2peak_ (mL/kg/min)	56.1 (53.7, 58.4)	58.8 (55.6, 61.9)	51.7 (49.0, 54.4)
Total MVPA (min)	536.7 (454.9, 618.4)	515.2 (404.1, 626.3)	571.7 (443.8, 699.5)
Type of primary endurance sport			
Middle-distance running	13 (26%)	10 (32.3%)	3 (15.8%)
Long-distance running	45 (90%)	27 (87.1%)	18 (94.7%)
Triathlon	5 (10%)	3 (9.7%)	2 (10.5%)
Swimming	8 (16%)	3 (9.7%)	5 (26.3%)
Cycling	12 (24%)	9 (29%)	3 (15.8)
Other	9 (18%)	7 (22.6%)	2 (10.5%)
Training distance (km/wk)			
15–25	11 (22%)	8 (25%)	4 (20%)
25–40	16 (32%)	9 (28%)	7 (35%)
>40	23 (46%)	15 (47%)	9 (45%)
Race/ethnicity			
White	35 (70%)	22 (69%)	15 (75%)
Black	1 (2%)	1 (3%)	0
Hispanic	2 (4%)	0	2 (10%)
Asian	7 (14%)	5 (16%)	2 (10%)
Middle Eastern/North African	3 (6%)	2 (6%)	1 (5%)
Other	2 (4%)	2 (6%)	0
Peak knee extensor torque (N/m)	262.3 (232.0, 292.7)	315.4 (284.4, 346.4)	177.4 (150.4, 204.4)
Maximum grip strength (kg)	43.5 (40.2, 46.8)	49.8 (46.6, 52.0)	33.4 (31.5, 35.4)
Vitamin D supplement	25 (50%)	16 (52%)	9 (47%)
Total BPAQ Score	24.03 (19.0, 29.1)	23.23 (18.0, 28.5)	25.38 (14.3, 36.5)
Cognitive restraint score (TFEQ)	32.3 (25.9, 38.6)	32.8 (23.7, 41.8)	31.5 (22.6, 40.4)
EDI-3 total score	13.2 (9.6, 16.8)	12.7 (8.2, 17.2)	14.0 (7.4, 20.6)

Data are presented as mean and 95% confidence interval for mean in parentheses; primary endurance sport, race/ethnicity, and vitamin D data are presented as number of participants and percentage in parentheses.

Abbreviations: BF, body fat; BPAQ, Bone Specific Physical Activity Questionnaire; EDI-3, Eating Disorder Inventory-3; FFM, fat-free mass; LBM, lean body mass; MVPA, moderate-to-vigorous physical activity; TFEQ, Three Factor Eating Questionnaire.

### pQCT and DXA

Tibial trabecular and cortical vBMD and area, and estimated bone strength results are summarized in Table 2. aBMD and *Z*-score results are summarized in Table 3. Out of 44 participants with an available aBMD Z-score (18 years or older), only 2 participants (5%) had an LS aBMD *Z*-score below −2.0. As defined by the ACSM Female Athlete Triad guidelines, active individuals with a *Z*-score of −1.0 are considered to have low BMD for age [4]. In this cohort, 13 participants (26%) had an LS aBMD *Z*-score below −1.0; 4 participants (9%) had an FN aBMD *Z*-score below −1.0; and 2 participants (5%) had a TH aBMD *Z*-score below −1.0.

**TABLE 2:** Tibial volumetric bone mineral density, area, and estimated bone strength, and calf muscle area and density in male and female endurance-trained individuals from peripheral quantitative computed tomography (pQCT).

Variable	All (*n* = 50)	Males (*n* = 31)	Females (*n* = 19)
**4%**			
Trabecular vBMD (mg/mm^3^)	260.7 (250.0, 271.4)	273.3 (258.9, 287.7)	240.2 (228.5, 251.9)
Trabecular area (mm^2^)	494.6 (470.5, 518.8)	533.2 (504.8, 561.5)	431.8 (405.2, 458.4)
BSI	137.0 (119.9, 154.1)	161.4 (137.8, 185.1)	97.1 (90.3, 104.0)
**38%**			
Cortical vBMD (mg/mm^3^)	1155.8 (1148.3, 1163.4)	1151.9 (1143.8, 1160.0)	1162.3 (1146.7, 1177.9)
Cortical area (mm^2^)	344.0 (326.2, 361.9)	366.5 (348.8, 384.3)	307.4 (274.9, 339.9)
SSI_p_ (mm^3^)	2027.5 (1890.5, 2164.5)	2256.8 (2109.9, 2403.7)	1653.4 (1481.5, 1825.4)
**66%**			
Cortical vBMD (mg/mm^3^)	1098.4 (1090.9, 1106.0)	1091.3 (1082.5, 1100.0)	1110.1 (1097.1, 1123.2)
Cortical area (mm^2^)	325.2 (308.6, 341.8)	357.0 (339.6, 374.4)	273.4 (258.2, 288.6)
SSI_p_ (mm^3^)	3258.5 (3010.7, 3506.2)	3722.3 (3462.7, 3981.9)	2501.8 (2256.7, 2746.8)
Muscle CSA (mm^2^)	7359.2 (6954.7, 7763.6)	7984.0 (7500.6, 8467.5)	6339.7 (5895.8, 6783.6)
Muscle density (mg/cm^3^)	79.1 (78.7, 79.4)	79.1 (78.7, 79.5)	79.0 (78.4, 79.6)

Data are presented as mean and 95% confidence interval for mean in parentheses.

Abbreviations: BSI, bone strength index; CSA, cross-sectional area; vBMD, volumetric bone mineral density; SSI_p_, polar stress-strain index.

**TABLE 3:** Whole-body and site-specific areal bone mineral density and available Z-scores in male and female endurance-trained individuals from dual-energy x-ray absorptiometry.

Variable	All (*n* = 50)	Males (*n* = 31)	Females (*n* = 19)
Total body aBMD (g/cm^2^)	1.25 (1.21, 1.29)	1.31 (1.26, 1.36)	1.16 (1.11, 1.20)
Total body *Z*-score	1.11 (0.80, 1.42)	1.27 (0.82, 1.72)	0.87 (0.48, 1.26)
Total body BMC (g)	2.80 (2.64, 2.96)	3.09 (2.90, 3.28)	2.33 (2.20, 2.45)
LS aBMD (g/cm^2^)	1.19 (1.15, 1.23)	1.22 (1.16, 1.28)	1.14 (1.08, 1.20)
LS *Z*-score	−0.08 (−0.43, 0.27)	0.10 (−0.39, 0.59)	−0.37 (−0.90, 0.16)
LS *Z*-score below −2.0	2 (4.55%)	1 (3.70%)	1 (5.88%)
LS *Z*-score below −1.0	13 (29.54%)	7 (25.93%)	6 (35.30)
FN aBMD (g/cm^2^)	1.10 (1.05, 1.14)	1.14 (1.08, 1.20)	1.03 (0.99, 1.07)
FN *Z*-score	0.22 (−0.08, 0.52)	0.38 (−0.07, 0.83)	−0.03 (−0.33, 0.27)
FN *Z-*score below −2.0	0 (0%)	0 (0%)	0 (0%)
FN *Z*-score below −1.0	4 (9.10%)	2 (7.40%)	2 (11.76%)
TH aBMD (g/cm^2^)	1.11 (1.07, 1.15)	1.15 (1.09, 1.21)	1.03 (1.00, 1.07)
TH *Z*-score	0.27 (0.03, 0.51)	0.30 (−0.07, 0.66)	0.23 (−0.05, 0.51)
TH *Z*-score below −2.0	0 (0%)	0 (0%)	0 (0%)
TH *Z*-score below −1.0	2 (4.55%)	2 (7.40%)	0 (0%)

Data are presented as mean and 95% confidence interval for mean in parantheses. *Z*−scores are presented as number of participants and percentage in parentheses; *Z−*scores are only available for 44 participants.

Abbreviations: aBMD, areal bone mineral density; BMC, bone mineral content; FN, femoral neck; LS, lumbar spine; TH, total hip.

### Macronutrients

**FIGURE 1:**
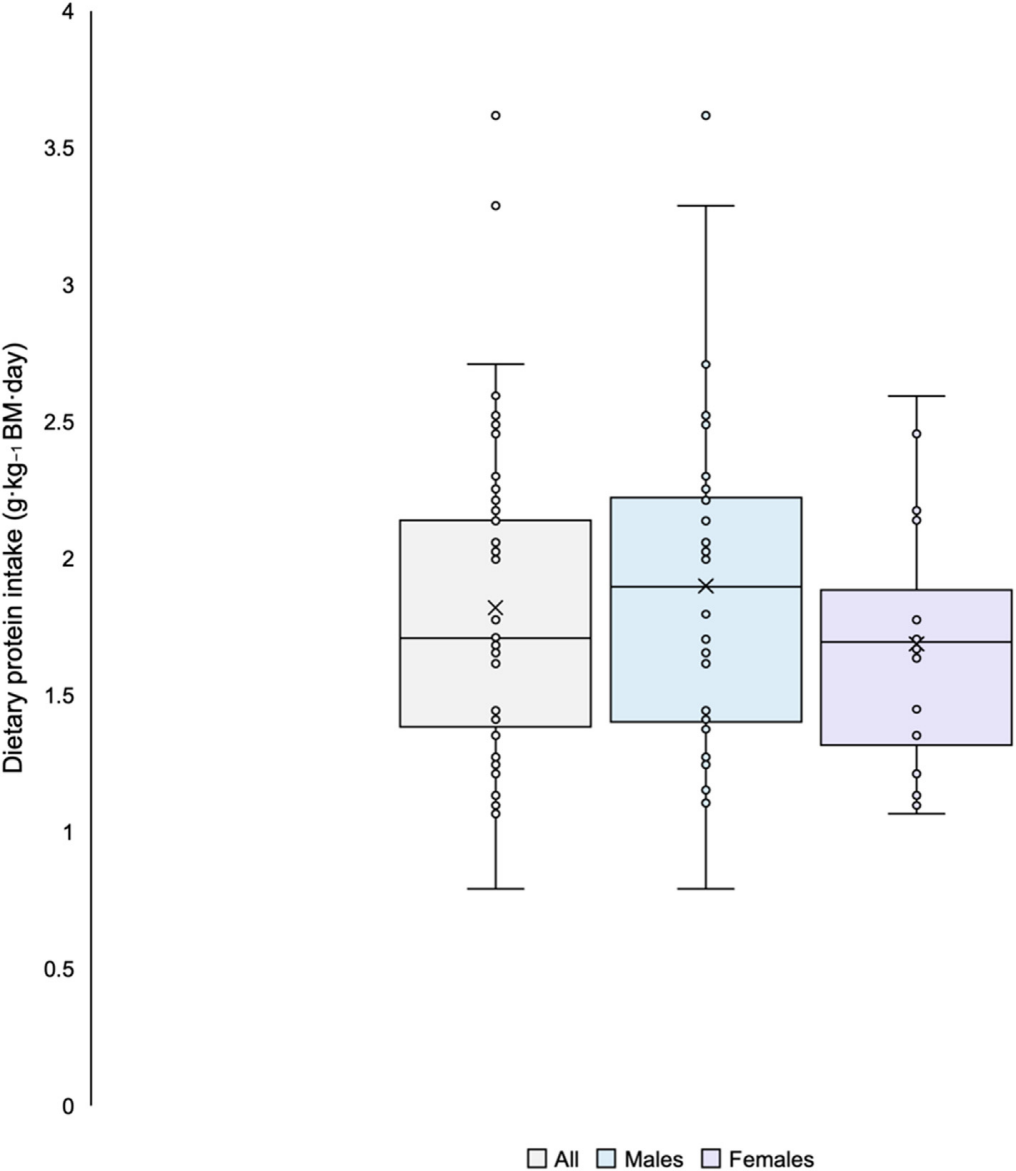
Plots of total dietary protein intake (grams per kilogram of body mass per day) of endurance-trained participants.

Two participants were excluded from the analyses because they did not complete any of the required dietary recalls. Mean daily consumption of dietary macronutrients and recommended intakes are presented in Table 4. Total dietary protein intake results are summarized in Figure 1 as box-and-whiskers plots. Only 3 males (10%) and 3 females (17%) did not meet the ACSM athlete dietary protein recommendations of ≥1.2 g/kg BM/d [30]. Mean dietary protein intake was 1.8 g/kg BM/d, which is well within the ACSM recommended intake range of 1.2–2.0 g/kg BM/d, and the mean percentage of kilocalories from protein was 19.2%. Eighteen participants (37.5%) consumed a dietary protein intake ≥2.0 g/kg BM/d. A large proportion of participants (81%) did not meet ACSM dietary carbohydrate (CHO) recommendations of 6–10 g/kg BM/d. Over 60% of participants were not consuming 20%–35% of kcals from dietary fat as suggested by the ACSM and the AMDR [30].

**TABLE 4:** Dietary energy and macronutrient intake of male and female endurance-trained participants from Automated Self-Administered 24-hour dietary recall.

	All (*n* = 48)		Males (*n* = 30)		Females (*n* = 18)		Recommended intake^1^^,^^2^^,^^3^
	Mean	% not meeting recommendations *n,* %	Mean	% not meeting recommendations *n,* %	Mean	% not meeting recommendations *n,* %	
Total dietary energy intake (kcal/d)	2675.3 (2455.1, 2895.6)	N/a	2905.1 (2624.6, 3185.6)	N/a	2292.5 (1986.3, 2598.7)	N/a	N/a
Dietary CHO (g/kg BM/d)	4.5 (3.9, 5.1)	39, 81.3%	4.4 (3.6, 5.2)	24, 80%	4.6 (3.8, 5.3)	15, 83.3%	6–10^1^
% kcals from CHO	44.7 (41.4, 48.0)	21, 43.8%	43.4 (38.3, 48.6)	15, 50%	46.8 (44.2, 49.3)	6, 33.3%	45%–65%^2^
Dietary PRO (g/kg BM/d)	1.8 (1.7, 2.0)	6, 12.5%	1.9 (1.7, 2.1)	3, 10%	1.7 (1.5, 1.9)	3, 16.7%	1.2^1^
% kcals from PRO	19.2 (17.5, 20.9)	0, 0%	20.1 (17.6, 22.5)	0, 0%	17.8 (15.8, 19.7)	0, 0%	10%–35%^2^
Dietary fat (g/d)	110.0 (97.9, 122.2)	N/a	121.4 (105.0, 137.7)	N/a	91.1 (75.8, 106.5)	N/a	N/a
% kcals from fat	36.5 (34.2, 38.9)	30, 62.5%	37.2 (33.7, 40.7)	19, 63.3%	35.4 (32.6, 38.2)	11, 61.1%	20%–35%^1^^,^^2^
Dietary calcium (mg/d)	1170.9 (1002.0, 1339.8)	20, 41.7%	1197.8 (956.2, 1439.4)	13, 43.4%	1126.0 (893.8, 1358.2)	7, 38.9%	1000^3^
Vitamin D (IU)	293.9 (208.2, 379.6)	43, 89.5%	337.1 (204.6, 469.7)	26, 86.7%	226.6 (144.7, 308.6)	17, 94.4%	600^3^

Data are presented as mean and 95% confidence interval for mean in parentheses. Two participants are excluded, 1 male and 1 female, for no data.

Abbreviations: AMDR, acceptable macronutrient distribution range; BM, body mass; CHO, carbohydrates; PRO, protein; RDA, recommended dietary allowance; N/a, not appicable.

1 Thomas et al., 2016. [30]

2 AMDR.

3 RDA.

### Associations between total dietary protein intake and bone and muscle parameters

Bivariate analyses between total dietary protein intake and measures of vBMD, SSI_p_, and BSI are in Table 5. Bivariate analyses with muscle density, area, and strength are in Table 6. In all participants, there were no significant correlations between total dietary protein intake and pQCT bone and muscle variables. However, total dietary protein intake was significantly correlated with LS (r = 0.42, *P =* 0.003), FN (r = 0.30, *P* = 0.043), and TH (r = 0.37, *P* = 0.009) aBMD. To explore the influence of sex on our findings, these relationships were stratified by sex (not shown), and we found the relationships were statistically significant in males but not females, and this is likely due to the smaller sample of females (*n* = 18).

**TABLE 5:** Pearson correlations for the relationships between dietary protein intake and bone properties from pQCT and DXA in all participants.

Total dietary protein intake (g/kg BM/d)	All (*n* = 48)	
	r	*P* value
**pQCT**		
4%		
Trabecular vBMD (mg/mm^3^)	0.163	0.268
BSI (mg^2^/mm^4^)	0.034	0.818
38%		
Cortical vBMD (mg/mm^3^)	−0.102	0.491
SSI_p_ (mm^3^)	−0.002	0.991
66%		
Cortical vBMD (mg/mm^3^)	−0.221	0.131
SSI_p_ (mm^3^)	0.080	0.587
**DXA**		
LS aBMD (g/cm^2^)	0.415∗	0.003∗
FN aBMD (g/cm^2^)	0.294∗	0.043∗
TH aBMD (g/cm^2^)	0.373∗	0.009∗

Data are presented as Pearson coefficient (r).

Abbreviations: aBMD, areal bone mineral density; BSI, bone strength index; CSA, cross-sectional area; DXA, dual-energy x-ray absorptiometry; FN, femoral neck; LS, lumbar spine; pQCT, peripheral quantitative computed tomography; SSI_p_, polar stress-strain index; TH, total hip; vBMD, volumetric bone mineral density. ∗denotes p<0.05.

**TABLE 6:** Pearson correlations for the relationship between dietary protein intake and muscle density, area, and strength in all participants.

Total dietary protein intake (g/kg BM/d)	All (*n* = 48)	
	r	*P* value
**pQCT muscle parameters (66%)**		
Muscle density (mg/cm^3^)	0.069	0.639
Muscle CSA (mm^2^)	0.167	0.256
**Muscle strength**		
Maximum peak torque (N/m)	0.095	0.526
Maximum grip strength (kg)	0.195	0.185

Data are presented as Pearson coefficient (r).

Abbreviations: CSA, cross-sectional area; pQCT, peripheral quantitative computed tomography.

Results of the multivariable regression analyses are in Table 7. In the unadjusted model, total dietary protein intake was significantly associated with LS aBMD (R^2^ = .188, F = 10.66, *P* = 0.002) and TH aBMD (R^2^ = .134, F = 7.09, *P* = 0.011). The models were adjusted for sex, LBM, MVPA, and calcium intake, and a positive association persisted only between total dietary protein intake and LS aBMD (β = 0.398, *P* = 0.009). Of all confounding variables, LBM was the only predictor that explained FN (*P* = 0.041) and TH (*P* = 0.049) aBMD.

**TABLE 7:** Regression model of total dietary protein intake on areal BMD in all participants.

	Total dietary protein (g/kg BM/d)				
	β (SE)	t value	*P* value	Model R^2^	Effect size (Cohen’s f^2^)
**LS aBMD (g/cm^2^)**					
Unadjusted	0.434 (0.032)	3.265	0.002∗	0.188	0.23
Adjusted	0.398 (0.034)	2.761	0.009∗	0.264	0.18
**FN aBMD (g/cm^2^)**					
Unadjusted	0.283 (0.036)	2.003	0.051	0.080	0.09
Adjusted	0.212 (0.035)	1.529	0.134	0.319	0.05
**TH aBMD (g/cm^2^)**					
Unadjusted	0.365 (0.035)	2.663	0.011∗	0.134	0.15
Adjusted	0.273 (0.035)	2.007	0.051	0.347	0.09

Unadjusted and adjusted for sex, LBM, MVPA, and calcium intake.

Effect size = Cohen’s f^2^ for linear models such that f^2^ ≥ 0.02, f^2^ ≥ 0.15, and f^2^  ≥ 0.35 are interpreted as small, medium, and large effect sizes, respectively [63].

Abbreviations: aBMD, areal bone mineral density; FN, femoral neck; LS, lumbar spine;

TH, total hip. ∗denotes p<0.05.

### Associations between protein intake from animal products and bone and muscle parameters

Bivariate analyses between protein intake from animal products and measures from pQCT, DXA, and muscle strength were conducted (Supplemental Tables 1 and 2). In all participants, there were significant correlations between protein intake from animal products and aBMD at the LS (r = 0.47, *P* < 0.001), FN (r = 0.54, *P* < 0.001), and TH (r = 0.54, *P* < 0.001) site. Significant correlations were also seen between protein intake from animal products and SSI_p_ at the 38% (r = 0.39, *P* = 0.008) and 66% sites (r = 0.44, *P* = 0.002), cortical vBMD at the 66% site (r = −0.34, *P* = 0.02), muscle CSA at the 66% site (r = 0.57, *P* < 0.001), maximum peak torque (r = 0.35, *P* = 0.019), and maximum grip strength (r = 0.42, *P* = 0.004). In the unadjusted regression models, protein intake from animal products was significantly associated with LS (R^2^ = .268, F = 16.15, *P* < 0.001), FN (R^2^ = .321, F = 20.83, *P* < 0.001), and TH (R^2^ = .346, F = 23.33, *P* < 0.001) aBMD. The models were adjusted for sex, LBM, MVPA, and calcium intake, and positive associations persisted with LS (β = 0.495, *P* = 0.007), FN (β = 0.378, *P* = 0.024), and TH (β = 0.435, *P* = 0.009) aBMD. Unadjusted regression models were conducted for SSI_p_ at the 38% (β = 0.377, *P* = 0.010) and 66% sites (β = 0.418, *P* = 0.004), cortical vBMD (β = −0.280, *P* = 0.059), and muscle CSA (β = 0.650, *P* < 0.001). Adjusted models revealed that muscle CSA remained associated with protein intake from animal products (β = 0.269, *P* = 0.031). LBM was the only significant predictor (*P* < 0.001) of 38% and 66% SSI_p_.

Total dairy product intake was extracted from the ASA24 variable coded “D_TOTAL” and was also analyzed against bone and muscle measures, but no significant relationships were found except with muscle CSA at the 66% site (r = 0.33, *P* = 0.02).

## Discussion

The current study explored the relationships between dietary protein intake and BMD, estimated bone strength, and muscle area, density, and strength in endurance-trained individuals. We found no associations between total protein intake and tibial vBMD, estimated bone strength, and muscle area, density, and strength. However, protein intake from animal products was significantly correlated with estimated bending strength (SSI_p_) at the 38% and 66% sites, cortical vBMD at the 66%, and tibial muscle CSA. We also found associations that suggest dietary protein intake may explain a small variance in LS aBMD, a finding consistent with the literature in non-athletic populations.

Numerous meta-analyses have established the positive influence of dietary protein intake on bone health in healthy [64] and clinical [29,65] populations. The current observational study explored this relationship to extend the evidence to endurance-trained individuals by measuring vBMD and bone strength using pQCT and aBMD using DXA. We did not find any relationships between total dietary protein intake and pQCT-derived bone parameters. This is somewhat surprising as it has been shown in prepubescent girls, older adults, and postmenopausal females that dietary protein intake is associated with pQCT- and high-resolution-pQCT (HR-pQCT) derived bone parameters [18,[66], [67], [68]]. One explanation may be that dietary protein intake is more beneficial to bone during growth and development and aging in contrast to our sample of healthy, highly active individuals with a mean dietary protein intake of 1.8 g/kg BM/d (90% of participants were achieving the minimum 1.2 g/kg BM/d ACSM recommendation for athletes). Results of this analysis could also differ if our sample had a larger range of protein intake (i.e., low vs. high protein intake) or age (i.e., adolescent athletes compared with master athletes). Our lack of findings between total dietary protein intake and pQCT measures, but a positive association with LS aBMD, may also suggest that the effects of dietary protein may differ by central and peripheral sites in this particular population. Although the tibia is more representative of bone stress injury risk and relevant to the loading induced by direct ground reaction forces and impacts, this region may be less influenced by nutrition compared to clinically significant DXA measures as it is well established that central skeletal sites (i.e., hip and LS) are significantly affected by energy deficiency and hypogonadism [[69], [70], [71], [72]]. The LS site is predominantly made up of trabecular bone, which is more sensitive to external stimuli and metabolic changes such as nutritional deficiencies due to the higher surface-to-volume ratio when compared to cortical bone [73,74]. Our analyses would benefit from the evaluation of other peripheral sites (i.e., radius, femur) to make stronger conclusions about site-specific differences in the relationship between protein intake and bone outcomes. Further, after adjusting the models for confounders, protein intake (total and from animal products) was no longer associated with other DXA and pQCT measures, and instead, the association with protein intake was accounted for by LBM. It is well established in healthy and clinical populations that LBM is a significant determinant of bone properties, particularly bone size and strength [75]. LBM has also been suggested to predict stress fracture risk in female military recruits [76] and runners [77]. The only pQCT measure that remained significantly associated with protein intake from animal products after adjusting for confounders was muscle CSA, and LBM still significantly contributed to explaining variance in muscle CSA (*P* < 0.001).

Another explanation may be due to the source of protein intake. As proteins (collagen and non-collagenous proteins) comprise nearly 50% of bone volume, amino acids are essential for intracellular and extracellular bone protein synthesis, calcium–phosphate balance, and bone turnover [25,78,79]. The constituents of dairy products (calcium, vitamin D, and dietary protein) are also strongly associated with bone health outcomes [24,80]. It has been shown in young adults and clinical populations that dietary protein intake from animal sources (including dairy) is associated with pQCT bone outcomes [18,66,68]. This has also been reported in young female runners, where animal protein intake was positively associated with prospective annual gains in whole-body BMD and BMC measured by DXA [20]. Although we could not directly derive animal and plant protein intake measures from the ASA24 recalls, we were able to extract values associated with protein consumed from animal products and total dairy intake. The analysis revealed no significant relationships between bone measures and total dairy intake, but protein intake from animal products was significantly correlated with SSI_p_ at the 38% and 66% sites and cortical vBMD and muscle CSA at the 66% site. Thus, while we found no associations with total protein, some pQCT measures were significantly correlated with protein intake from animal products. Although these findings indicate weak relationships (r = 0.1–0.5) and were not significant after adjusting for confounders, they warrant a future analysis between protein amount and source and bone macro/microarchitecture, as well as finite element-based analyses of bone strength (i.e., pQCT, HR-pQCT). A larger sample may also yield greater statistical power to detect associations, which may translate to more relevant interpretations, especially because pQCT measures such as SSI_p_ (a surrogate of bone strength in response to bending and torsional strain) are often higher in long-distance runners than controls despite lower cortical vBMD [7].

As a result of the nature of low-to-moderate impact loading forces and dietary energy requirements, endurance athletes’ bone health is seemingly inferior to athletes participating in other sports, particularly higher impact sports, as noted by lower aBMD at clinically relevant sites (LS, FN) in young and master athletes [3,12,81]. The link between dietary protein intake and DXA-measured aBMD has been previously demonstrated in healthy adults, older males and females, endurance runners, young girls, and clinical populations [17,18,20,26,82,83]. However, this has not been consistently reported in non-athletic (see Darling review [84]) or athletic populations. A recent study found high protein intake was a negative predictor of LS aBMD in adolescent soccer players (although they only used one 24-h dietary recall to measure dietary intake) [85]. In oligomenorrheic endurance athletes, vegetable protein intake has been reported to be inversely associated with LS aBMD *Z-*score [23]. In this cohort, primarily made up of long-distance runners, we found that total protein intake was associated with LS aBMD after adjusting for known confounders, and protein intake from animal products was associated with all aBMD sites. A similar study that compared nutritional intake variables to bone outcomes in endurance runners was conducted by Nieves et al. [20], but unlike their findings, we found significant associations between total dietary protein intake and LS aBMD. The differing results are interesting because their sample presented with lower LS aBMD, and our cohort had 6 females (32%) exhibiting low BMD for age at the LS [4]. Their findings only persisted in females with menstrual irregularities, and although we did not stratify our results this way, nearly 60% of our female cohort reported their menses had stopped for 3 consecutive months or longer in the past. Additionally, their study design was prospective compared to our cross-sectional measures; therefore, direct comparisons are difficult to make.

There is well established evidence that supports the positive effects of muscle function on bone strength in healthy and athletic populations [38,86,87]. Through mechanical and metabolic processes, muscle and bone form a functional relationship, and by targeting muscle size, density, and strength (through contractile forces and impact loading), bone can interdependently alter size, density, and strength [88,89]. Dietary protein intake stimulates increased rates of skeletal muscle protein synthesis after endurance exercise, which may lead to increases in muscle size and strength [[90], [91], [92]]. We found that protein intake from animal products was associated with muscle CSA (*P <* 0.001) and tests of peak upper- (*P* = 0.004) and lower-body (*P* = 0.02) strength. However, we did not find any associations between total dietary protein intake and each of muscle density, muscle CSA, and strength. This may be due to our sample’s relatively high protein intake, thus rendering it difficult to detect significant relationships. The primary analysis of this study will comprehensively explore the associations between muscle factors and bone strength.

This study includes several strengths. Primarily, we were able to investigate the relationship between dietary protein intake and bone properties, not only with DXA but also with “true” vBMD and “bone quality” parameters by pQCT. This study also included male and female endurance-trained individuals and provides accounts of nutritional intake compared to current standards for athletes, which is valuable information in the field of sports nutrition [30]. Limitations of this study primarily concern the cross-sectional design (no causal effects can be inferred) and relatively small sample size, which prevented us from conducting analyses stratified by sex, which would be very insightful, especially given the greater risk of compromised bone health in female athletes. Two participants did not complete any dietary recalls, thus further reducing our sample size. Second, we could not directly extract dietary protein source data, thus limiting our investigation to self-reported total dietary protein intake and protein intake associated with animal product consumption. A future study would benefit from analysis by total, animal, and vegetable-origin protein. Total protein intake range was small (1.8 ± 0.57) (only 6 participants were not meeting the ACSM recommendations), and perhaps our results may differ if the range was larger. The variability of protein intake by training day or resting day was also not examined, which may have provided additional insight. Third, self-reported 24-h dietary recalls are prone to underestimating energy intake and day-to-day variability; however, we used repeated measures, including 2 weekdays and 1 weekend day, to help limit this bias [93,94]. The recall assessment tool reduces the likelihood of the participants altering their dietary intake in anticipation of recording due to its retrospective method. Our sample did not include any participants with a current eating disorder, and thus, participants likely did not have clinically low energy intake. Lastly, the sample, although consisting of predominantly long-distance runners, included various types of endurance-trained individuals from highly trained (varsity/national level) to recreationally active individuals, and therefore, the interpretation of our results cannot be generalized to one athlete group. We also did not capture a large proportion of individuals at higher risk for bone fragility, with few participants exhibiting low BMD for age; however, we did capture an at-risk subgroup with 6 females presenting with *Z*-scores below −1.0 [4].

In conclusion, total dietary protein intake was not associated with vBMD and estimated bone strength (SSI_p_ and BSI) and muscle CSA, density, and strength in male and female endurance-trained individuals. However, we did find that protein intake from animal products was correlated with SSI_p_ at the 38% and 66% sites, cortical vBMD at the 66% site, and muscle CSA. Further, in accordance with findings derived from non-athlete populations, our findings suggest total dietary protein intake and protein intake from animal products may explain a small variance in LS aBMD independent of sex, LBM, MVPA, and calcium intake. Future longitudinal studies are needed to investigate the independent contribution of total dietary protein intake and other sources of protein on bone and muscle measures in athletic populations, especially those at higher risk of bone fragility.

## Author contributions

The authors’ responsibilities were as follows – JCG was responsible for the research design and funding acquisition; JL, AS, SG, SF, and JRL conducted research; SG analyzed the data; SG wrote the manuscript with critical review from JCG, JL, AS, SF, JRL, TCV, and AJ; SG and JCG had primary responsibility for final content. All authors read and approved the final manuscript.

## Funding

JCG is the recipient of a Fonds de recherche du Québec (FRQS) Chercheurs-boursiers Research Scholar Award (Junior 1) and Natural Sciences and Engineering Research Council (NSERC) Discovery Grant. SG is the recipient of a Réseau de recherche en santé buccodentaire et osseuse (RSBO) research fellowship award.

## Conflict of interest

The authors report no conflicts of interest.
