# Supplementary material for: Dietary Protein Intake and Its Associations With Bone Properties Using Peripheral Quantitative Computed Tomography and Dual-Energy X-Ray Absorptiometry in Endurance-Trained Individuals
Source: Curr Dev Nutr. 2025 May 9;9(6):107459. doi: 10.1016/j.cdnut.2025.107459 (PMC12173609; doi:10.1016/j.cdnut.2025.107459)
Supplement: Multimedia component 1 [file mmc1.docx]

**SUPPLEMENTARY TABLE 1.** Regression model of protein intake from animal products on areal BMD in all participants.

|  | **Total of meat, poultry, seafood, organ meat, and cured meat (oz. eq.)** | | | | |
| --- | --- | --- | --- | --- | --- |
|  | **β (SE)** | **t value** | ***p* value** | **Model R^2^** | **Effect size (Cohen’s f^2^)** |
| **LS aBMD (g/cm^2^)** |  |  |  |  |  |
| Unadjusted | 0.518 (0.003) | 4.019 | **<0.001** | **0.268** | 0.36 |
| Adjusted | 0.495 (0.004) | 2.828 | **0.007** | **0.292** | 0.23 |
| **FN aBMD (g/cm^2^)** |  |  |  |  |  |
| Unadjusted | 0.567 (0.003) | 4.564 | **<0.001** | **0.321** | 0.47 |
| Adjusted | 0.387 (0.004) | 2.341 | **0.024** | **0.395** | 0.19 |
| **TH aBMD (g/cm^2^)** |  |  |  |  |  |
| Unadjusted | 0.589 (0.003) | 4.380 | **<0.001** | **0.346** | 0.53 |
| Adjusted | 0.435 (0.004) | 2.768 | **0.009** | **0.430** | 0.25 |

Unadjusted and adjusted for sex, lean body mass (LBM), moderate-to-vigorous physical activity (MVPA), and calcium intake. aBMD = areal bone mineral density; LS = lumbar spine; FN = femoral neck; TH = total hip. Effect size = Cohen’s f^2^ for linear models such that f^2^ ≥ 0.02, f^2^ ≥ 0.15, and f^2^  ≥ 0.35 are interpreted as small, medium, and large effect sizes, respectively [63].

**SUPPLEMENTARY TABLE 2.** Regression model of protein intake from animal products on pQCT measures in all participants.

|  | **Total of meat, poultry, seafood, organ meat, and cured meat (oz. eq.)** | | | | |
| --- | --- | --- | --- | --- | --- |
|  | **β (SE)** | **t value** | ***p* value** | **Model R^2^** | **Effect size (Cohen’s f^2^)** |
| **38% SSI_p_** |  |  |  |  |  |
| Unadjusted | 0.377 (11.163) | 2.700 | **0.010** | 0.142 | 0.17 |
| Adjusted | -0.138 (10.620) | -1.038 | 0.306 | 0.592 | -0.02 |
|  |  |  |  |  |  |
| **66%**  **Cortical vBMD** |  |  |  |  |  |
| Unadjusted | -0.280 (0.688) | -1.938 | 0.059 | 0.079 | 0.09 |
| Adjusted | -0.113 (0.896) | -0.600 | 0.552 | 0.178 | 0.02 |
| **SSI_p_** |  |  |  |  |  |
| Unadjusted | 0.418 (19.991) | 3.048 | **0.004** | 0.174 | 0.21 |
| Adjusted | -0.063 (17.621) | -0.523 | 0.604 | 0.663 | -0.17 |
|  |  |  |  |  |  |
| **Muscle CSA** |  |  |  |  |  |
| Unadjusted | 0.650 (27.981) | 5.681 | **<0.001** | 0.423 | 0.73 |
| Adjusted | 0.269 (29.436) | 2.232 | **0.031** | 0.664 | 0.07 |

Unadjusted and adjusted for sex, LBM, MVPA, and calcium intake. vBMD = volumetric bone mineral density; SSI_p_ = polar stress-strain index; CSA = cross sectional area. Effect size = Cohen’s f^2^ for linear models such that f^2^ ≥ 0.02, f^2^ ≥ 0.15, and f^2^  ≥ 0.35 are interpreted as small, medium, and large effect sizes, respectively [63].
